# Study on the Loss and Characteristics of Giant Magnetostrictive Transducers

**DOI:** 10.3390/mi16090982

**Published:** 2025-08-26

**Authors:** Qiang Liu, Xiping He, Weiguo Wang, Yanning Yang

**Affiliations:** 1 School of Physics and Electronic Information, Yan’an University, Yan’an 716000, China; wwgissp@126.com (W.W.); yadxyyn@163.com (Y.Y.); 2 School of Physics and Information Technology, Shaanxi Normal University, Xi’an 710119, China

**Keywords:** giant magnetostrictive transducer, output amplitude, stress distribution, magnetic energy loss, simulation calculation

## Abstract

The purpose of this work is to enable the giant magnetostrictive transducer to work efficiently. In this work, the finite element method was used to carry out a dynamic analysis and magnetic analysis of the transducers of GMM rods with different structures, and the transducers of three structural rods were developed, and the output amplitude and impedance of the three transducers were experimentally tested. The results show that the stress of the rod near the end of the tail mass was larger than that near the end of the head mass. The eddy current and hysteresis losses of the transducer were mainly concentrated on the outer diameter surface of the rod, near the cutting slit, and near the connection between the slices. In addition, there is a certain amount of eddy current loss on the magnetic conductor, permanent magnet, and coil. In the transducer with the untreated rod, the resistance and inductance were the smallest. The inductance of the transducers with the sliced rods were greater than those in the transducers with the slit rods. The transducer with the untreated rod has the highest resonant frequency and the smallest output amplitude, the resonant frequency of the transducers with the sliced rods was lower than that of the transducers with the slit rods, while the output amplitude of the transducers with the sliced rods was greater than that of the transducers with the slit rods. The simulated values of the resonant frequency, output amplitude, resistance, and inductance of the transducers of the three structural rods were basically consistent with the tested values.

## 1. Introduction

Ultrasonic waves are widely applied in areas such as agriculture, industry, environmental preservation, and medical science. High-frequency electrical signals are converted into the required ultrasonic vibrations by the transducer, which are the primary components within the ultrasonic vibration system. At present, piezoelectric transducers and magnetostrictive transducers are the most widely employed in industrial applications [1]. When compared with piezoelectric ceramics, Giant Magnetostrictive Materials (GMMs) exhibit a high energy density, large magnetostrictive coefficient, high thermal conductivity, good heat dissipation, strong load capacity, no overheating failure, fast response speed, and lower sound velocity; its size is one-third smaller than that of a piezoelectric transducer of the same frequency [2]. These characteristics determine that GMMs are exceptional functional materials for developing high-power, large-amplitude, and wideband ultrasonic processing systems. GMMs’ outstanding dynamic performance render them suitable for applications in areas like sensors, transducers, loudspeakers, and vibration energy harvesting [3,4,5,6].

A giant magnetostrictive transducer converts electrical energy into mechanical vibration, while also producing a thermal power loss; this phenomenon is especially pronounced in high-frequency magnetic fields, resulting in a rapid temperature rise in the transducer. Thermal power loss can alter the magnetostrictive coefficient of GMMs, consequently lowering the working efficiency of the transducer [7]. Rising temperatures may also result in a drop in both the bandwidth and resonant frequency of the giant magnetostrictive transducer [8]. Li et al. proposed a magnetic energy loss model of GMMs according to the loss separation theory, and designed a thermal control system to optimize energy transmission and thermal dissipation, and enhance the performance of the transducer [9]. Zhao et al. proposed a forced water-cooling control method to keep the GMM of the giant magnetostrictive actuator at a stable temperature, and the temperature of the actuator under different excitation currents and water flow rates was calculated by finite element software, and the study confirmed the efficacy of this thermal control method through experiments [10]. Cooling or dissipating heat from the transducer can reduce its operating temperature, enabling it to operate stably, but the redundant heat produced reduces the efficiency of the transducer. Another method is to design the magnetic circuit configuration of the transducer to minimize losses and heat generation.

Liu et al. proposed a giant magnetostrictive actuator made of multi-layer silicon steel, which reduces the eddy current losses in the magnetic circuit component and enhances the vibration characteristics of the actuator [11]. Lan et al. sliced the permanent magnet of the magnetostrictive transducer, which reduced the eddy current loss in the transducer and suppressed the temperature increase during long-term operation [12]. Kaleta et al. proposed a method for the production of magnetostrictive composites containing epoxy resins and powdered GMMs, and compared the magnetostrictive composites with solid GMMs. The resistivity of GMM composites increases and the eddy current loss decreases, but its magnetostriction is 45% lower than that of solid GMMs [13]. He et al. proposed a geometric calculation approach for determining the number of radial slots in GMM rods, which saved on design costs [14]. Teng et al. developed a giant magnetostrictive transducer, and proposed a structure with digital slots on the GMM rod. Compared to uncut rods, the eddy current loss of rods featuring this digital slot structure was reduced by three-quarters [15]. Cao et al. developed a model for the eddy current loss in axially cut GMM rods, and found that axial cutting can effectively reduce losses by interrupting the eddy current routes compared with radial slitting [16]. Huang et al. fabricated various square rings of GMMs to study the changes in magnetic energy loss at various magnetic flux density amplitudes and frequencies [17]. Li et al. used Ansoft Maxwell to simulate the eddy currents of GMM rods with different structures; the vibration performance of transducers with a slit rod and sliced rod was studied using the piezomagnetic piezoelectric analogy method and experimental tests [18]. Six structural GMM rods were previously proposed, and the vibration performances and losses of several structural rods were studied [19,20]. Compared with GMM rods, the structure of the transducer is more complex, and there are more factors affecting the loss and vibration characteristics of the transducer. However, there is no deep study on the loss and vibration properties of the transducer. It is of great importance to investigate the output characteristics and loss of transducers to improve their vibration performance and expand their application fields [21].

In this paper, COMSOL Multiphysics 5.4 was used to simulate the dynamics of giant magnetostrictive transducers with different structures of GMM rods, and the losses, resistances, and inductances of the transducers with different structural rods were studied. Transducers with three types of structural rods were designed and manufactured, and their impedance and output amplitude were experimentally tested.

## 2. Structure of the Transducer

**Figure 1:**
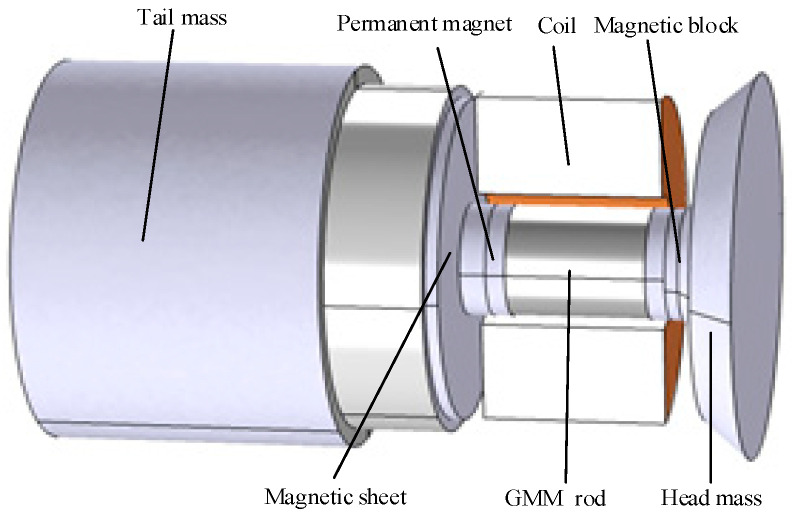
Schematic diagram of the giant magnetostrictive transducer.

Figure 1 presents a schematic diagram of the giant magnetostrictive transducer. The appropriate pre-stress was applied to the GMM rod by setting the pre-stressed bolt, and each component of the transducer was fixed. Permanent magnets were positioned on either end of the GMM rod to supply a bias magnetic field for the transducer. The high-frequency electrical signal generated by the ultrasonic generator excites the excitation coil of the transducer, thereby producing a high-frequency alternating magnetic field. An alternating magnetic field causes the GMM rod to create ultrasonic vibrations. The tail mass had a radius of 31 mm, and a length of 62.5 mm; the magnetic block measured 5 mm in length with a radius of 9 mm; the permanent magnet was 3 mm long and 9 mm in radius; the GMM rod featured a length of 21 mm and a radius of 9 mm; and the length of the head mass was 7 mm, the small end radius was 25 mm, and the large end radius was 31 mm.

## 3. Finite Element Calculation

Within the finite element software, the magnetic circuit analysis is based on Maxwell’s equations, whose differential form is expressed as follows:

(1) $$\nabla \times H=J+\frac{\partial D}{\partial t}$$

(2) $$\nabla \times E=-\frac{\partial B}{\partial t}$$

(3) ∇⋅D=ρ

(4) ∇×B=0

where ***H*** denotes the magnetic field strength, ***D*** represents the electric displacement, ***E*** stands for the electric field strength, ***B*** indicates the magnetic flux density, ***J*** refers to the current density, and *ρ* signifies the charge density.

The giant magnetostrictive transducer was simulated and computed in COMSOL Multiphysics 5.4 by utilizing the magneto–mechanical coupling characteristic of GMMs. The relationship between stress *S*, strain *ε*, magnetic flux density *B*, and magnetic field strength *H* is expressed in the stress-magnetization form:

(5) $$S=c_{H}\varepsilon -e_{HS}^{T}H$$

(6) $$B=e_{HS}\varepsilon_{e1}+\mu_{0}\mu_{rS}H$$

where *μ*_0_ denotes the permeability of the vacuum, *c_H_* represents the stiffness measured under a constant magnetic field strength, and *μ_rS_* stands for the relative permeability measured at a constant strain. *e_HS_* is the piezomagnetic coupling matrix.

For the electromechanical coupling problem in COMSOL Multiphysics, the governing equations for the finite element are as follows:

(7) $$\left[\begin{array}{cc} \left[M\right] & \left[0\right]\\ \left[0\right] & \left[0\right] \end{array}\right]\left[\begin{array}{c} \left[\ddot{u}\right]\\ \left[\ddot{A}\right] \end{array}\right]+\left[\begin{array}{cc} \left[C\right] & \left[0\right]\\ \left[0\right] & \left[0\right] \end{array}\right]\left[\begin{array}{c} \left[\dot{u}\right]\\ \left[\dot{A}\right] \end{array}\right]+\left[\begin{array}{cc} \left[K\right] & \left[K_{m}\right]\\ {\left[K_{m}\right]}^{T} & \left[K_{\mu }\right] \end{array}\right]\left[\begin{array}{c} \left[u\right]\\ \left[A\right] \end{array}\right]=\left[\begin{array}{c} \left[F\right]\\ \left[\phi \right] \end{array}\right]$$

where [*M*] represents the mass matrix, [*C*] denotes the damping matrix, [*K*] stands for the stiffness matrix, [*u*] is the structural displacement vector, [*F*] refers to the load force vector, [*A*] signifies the number of ampere-turns of excitation applied to the GMM, $\left[K_{m}\right]$ indicates the magnetostrictive coupling matrix, $\left[K_{\mu }\right]$ represents the magnetic permeability matrix, and [ϕ] denotes the magnetic flux passing through the cross-section.

In SolidWorks 2012, models of giant magnetostrictive transducers with GMM rods of different structures were built, which were then imported into COMSOL Multiphysics 5.4 for a simulation analysis. Table 1 shows the material parameters of each component of the transducer. According to reference [22], the piezomagnetic constant matrix, magnetic permeability matrix, and elastic constant matrix of the GMM rod were obtained.

**Figure 2:**
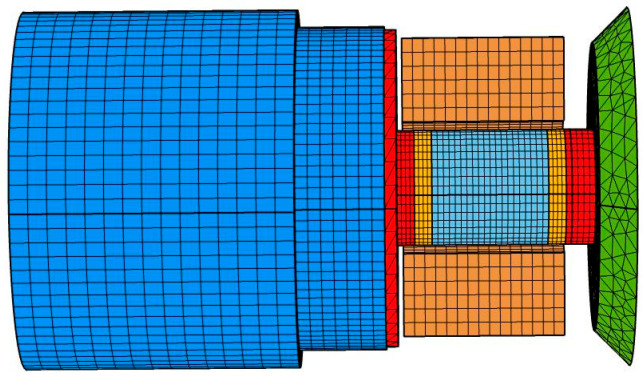
Grid diagram of transducer with untreated rod.

The magnetic field and solid mechanics were selected in the software, the coil has 350 turns, its wire model was configured as homogenized multiple-turns, its coil type was set to numeric, and the voltage was 100 V, and the dynamic analysis of the transducers with different structural rods was performed. Figure 2 shows the grid diagram of the transducer with the untreated rod: the total number of volume meshes and nodes were 48,277 and 26,110, respectively, and the air domain was relatively large, so the air domain outside the transducer was not displayed.

**Figure 3:**
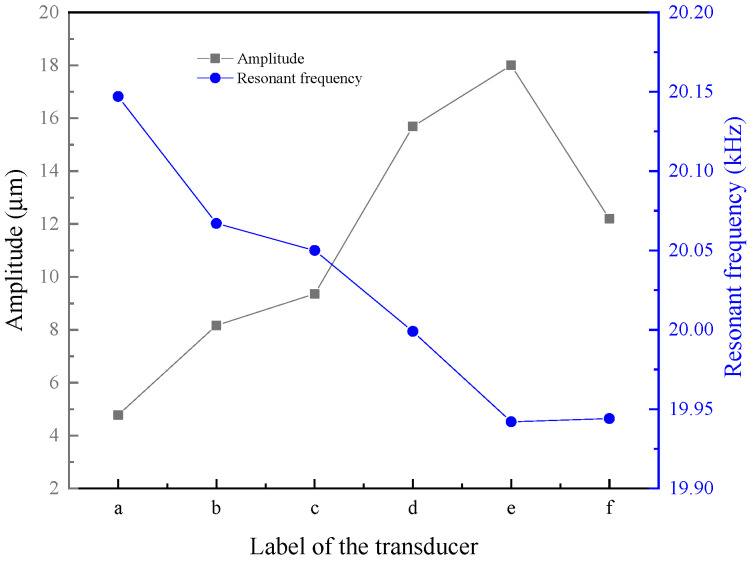
Output amplitude and resonant frequency of transducers with different structural rods. ((a) Untreated (b) Radial slit (c) Radial cut and bonded (d) Sliced and grooved (e) Slice treatment (f) Sliced at both ends).

Figure 3 shows the resonant frequency and output amplitude of transducers with different structural rods. Based on this figure, the resonant frequency of the transducer with the untreated rod was the largest, and the output amplitude was the smallest; the resonant frequency of the transducer with the sliced rods (Figure 3d–f) was lower than that of the transducer with the slit rods (Figure 3b,c), while the output amplitude of the transducer with the sliced rods was larger than that of the transducer with the slit rods; and the resonant frequency of the transducer with the slice treatment rod (Figure 3e) was the smallest, and the output amplitude was the largest.

**Figure 4:**
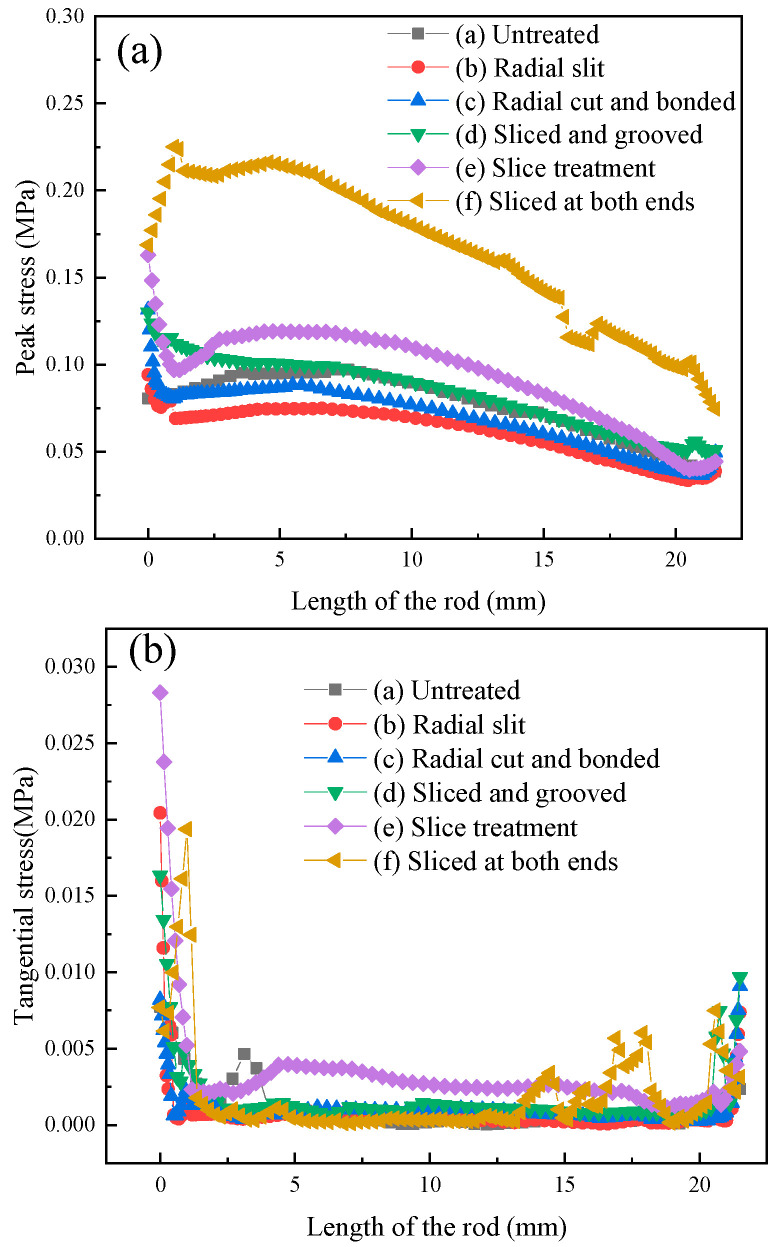
Stress of different structural rods: (**a**) peak stress, and (**b**) tangential stress.

Figure 4 shows the axial stress distribution of different structural rods in the transducer. The axial peak stress of the radial slit rod was the smallest, while the axial peak stress of the sliced rod at both ends was the largest. Both the peak stress and tangential stress of the rod close to the tail mass end were greater than those near the head mass end. The tangential stress of the slice treatment rod was the highest, while that of the untreated rod was the lowest. The reason is that, after the slicing treatment of the Terfenol-D rod, the magnetic field distribution inside the rod was more uniform, and the magnetostriction strain of the rod was larger, resulting in higher peak stress and tangential stress of the sliced rods. To improve the working efficiency of the transducer, the ultrasonic vibration energy generated by the Terfenol-D rod to radiate out from the head mass, the head mass is usually made of a material with low density, while the tail mass is made of a material with high density. The ultrasonic vibration energy generated by the Terfenol-D rod at the end close to the tail mass is not easy to radiate out, which leads to greater stress at the end near the tail mass.

When the transducer is working, the peak stress and tangential stress of the sliced rods should be less than the allowable stress, reducing the stress concentration of the rod, and suppress the generation and spread of microcracks. If the rod has microcracks, the long-term operation of the transducer will accelerate the spread of microcracks, and, when the microcracks extend to a certain extent, the rod will fracture, resulting in the failure of the transducer.

**Figure 5:**
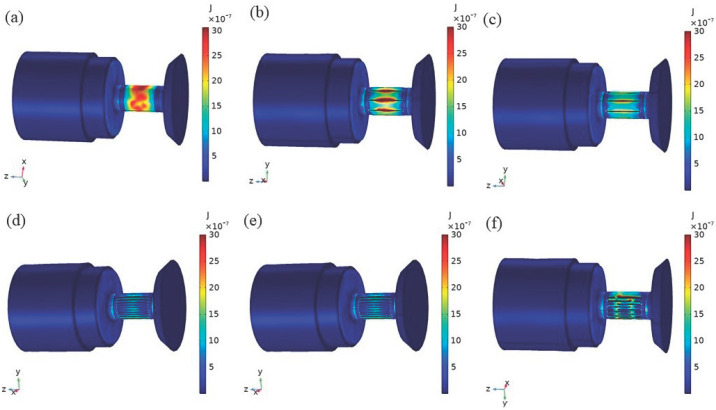
Eddy current loss distribution of transducers with several structural rods. (**a**) Untreated (**b**) Radial slit (**c**) Radial cut and bonded (**d**) Sliced and grooved (**e**) Slice treatment (**f**) Sliced at both ends.

The magnetic analysis of the transducers with structural rods was performed in COMSOL Multiphysics 5.4. Figure 5 shows the eddy current loss distribution of transducers with several rods over a period of time. The rod has the greatest eddy current loss, the magnetic sheets and magnetic blocks also have a certain amount of eddy current loss, the eddy current loss on the permanent magnets was relatively small, and the eddy current losses was minimized in the head mass and tail mass. The eddy current loss of the untreated rod transducer (Figure 5a) was mainly concentrated on the rod’s outer diameter surface, the eddy current loss of the transducer of the slit rods (Figure 5b,c) was mainly concentrated near the slit of the on the rod’s outer diameter surface, and the eddy current loss occurring on the external diameter surface of the transducer of the sliced rods (Figure 5e,f) was relatively small.

**Figure 6:**
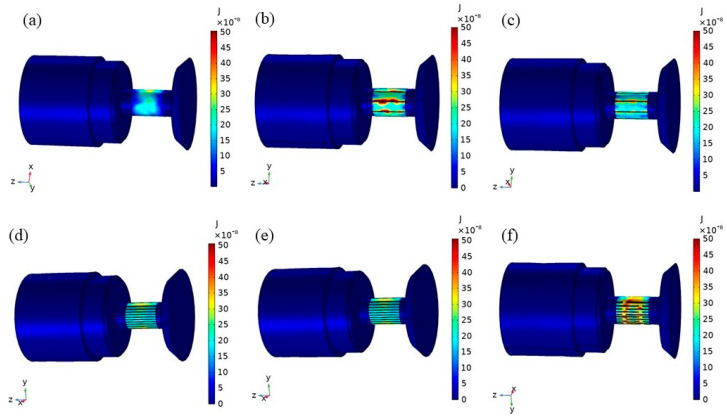
Hysteresis loss distribution of transducers with several structural rods. (**a**) Untreated (**b**) Radial slit (**c**) Radial cut and bonded (**d**) Sliced and grooved (**e**) Slice treatment (**f**) Sliced at both ends.

Figure 6 shows the hysteresis loss distribution of transducers with several structural rods over a period of time. The hysteresis loss occurring in the rod was the most significant, while the hysteresis loss of other parts of the transducer was relatively small. The hysteresis loss of the transducer of the untreated rod (Figure 6a) was concentrated on the external diameter surface of the rod; and the hysteresis loss of the transducer of the slit rods (Figure 6b,c) was mainly concentrated near the slit on the rod’s outer diameter surface. There is a certain amount of hysteresis loss near the joint of the slices in the transducer with sliced rods at both ends (Figure 6f).

**Figure 7:**
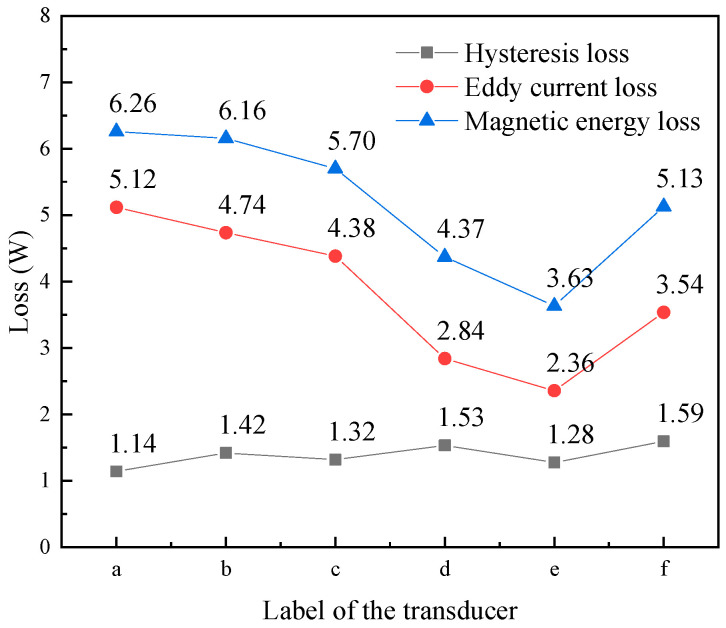
Magnetic energy loss of different structural rods. ((a) Untreated (b) Radial slit (c) Radial cut and bonded (d) Sliced and grooved (e) Slice treatment (f) Sliced at both ends).

The head mass and tail mass had relatively little eddy current loss, and its loss was ignored. Figure 7 shows the total magnetic energy loss of the rod in transducers with different structural rods. Magnetic energy loss includes hysteresis loss and eddy current loss. According to this figure, the eddy current loss was largest in the untreated rod (Figure 7a), whereas its hysteresis loss was the smallest; compared with the slit rods (Figure 7b,c), the sliced rods (Figure 7d,e) showed a lower eddy current loss; while, conversely, the hysteresis loss of the slit rods (Figure 7b,c) was smaller than that of the sliced rods (Figure 7d,f).

**Figure 8:**
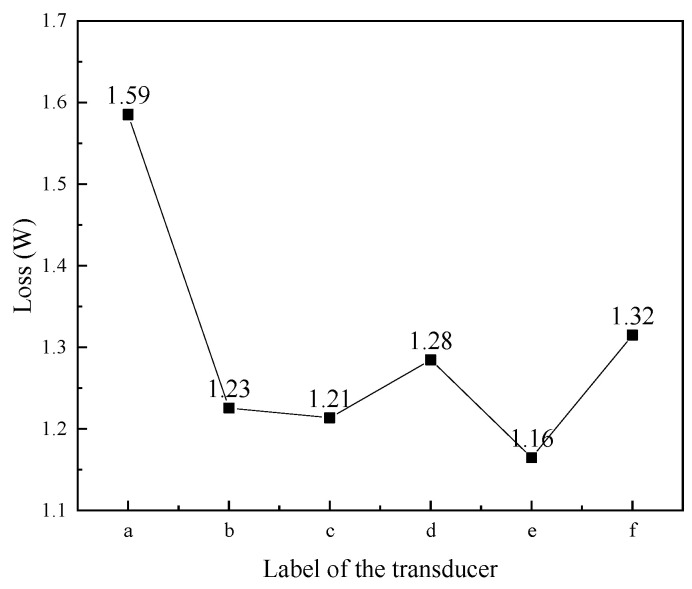
Eddy current loss of the magnetic conductor in transducers with different structural rods. ((a) Untreated (b) Radial slit (c) Radial cut and bonded (d) Sliced and grooved (e) Slice treatment (f) Sliced at both ends).

Figure 8 shows the total eddy current loss of the magnetic conductor in transducers with different structural rods. Magnetic conductors include magnetic blocks and magnetic sheets. The results showed that the eddy current loss of the magnetic conductor in the transducer with the untreated rod (Figure 8a) was the highest, whereas the magnetic conductor in the transducer with slice treatment rod (Figure 8e) was the lowest. The magnetic conductor was located at both ends of the Terfenol-D rod. The structure of the rod affects the axial magnetic field distribution of the coil, and also has an impact on the magnetic field distribution inside the magnetic conductor. The untreated rod has a serious skin effect under the excitation of the high-frequency magnetic field, and its eddy current loss was large, which also leads to the large eddy current loss of the magnetic conductor of the transducer of the untreated rod. However, the magnetic field inside the slice treatment rod was relatively uniform, resulting in a small eddy current loss, and the eddy current loss in the magnetic conductor of the transducer of the slice treatment rod was also small.

**Figure 9:**
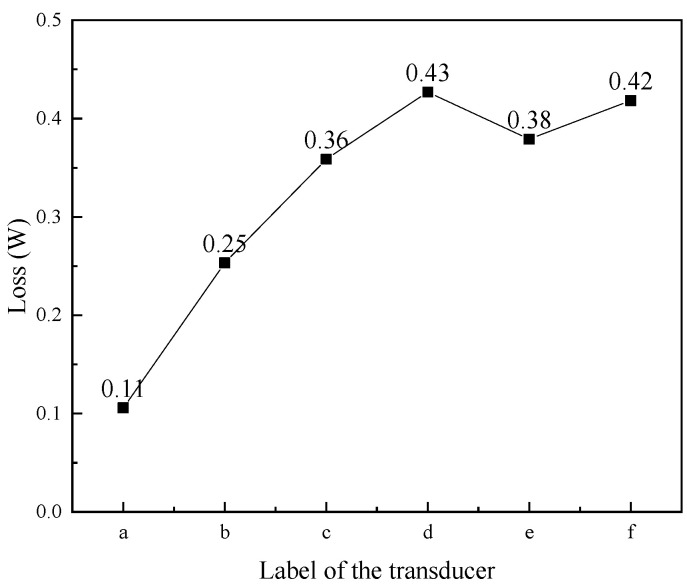
Eddy current loss of the permanent magnet in transducers with different structural rods. ((a) Untreated (b) Radial slit (c) Radial cut and bonded (d) Sliced and grooved (e) Slice treatment (f) Sliced at both ends).

Figure 9 shows the total eddy current loss of the permanent magnet in transducers with different structural rods. The permanent magnet in the transducer with an untreated rod (Figure 9a) exhibited the lowest eddy current loss; the eddy current loss of the permanent magnet in the transducers with sliced rods (Figure 9d–f) was larger than that in those with slit rods (Figure 9b,c). Permanent magnets provide a bias magnetic field for the Terfenol-D rod to eliminate the frequency doubling phenomenon. The eddy current loss of the permanent magnet in the transducer with the sliced rods was relatively large, and the eddy current loss will spread out in the form of heat. When the transducer is working, if the temperature of the permanent magnet is too high, it will cause demagnetization, resulting in the transducer failing to work normally.

**Figure 10:**
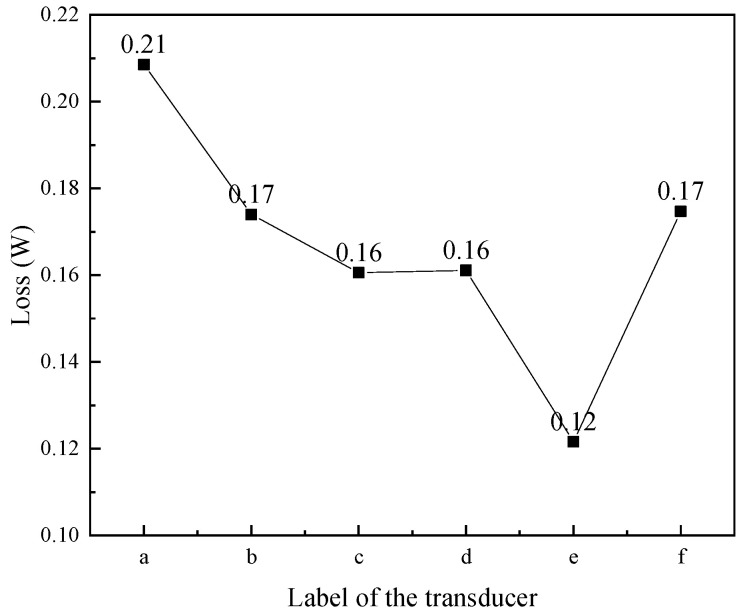
Eddy current loss of coils in transducers with different structural rods. ((a) Untreated (b) Radial slit (c) Radial cut and bonded (d) Sliced and grooved (e) Slice treatment (f) Sliced at both ends).

Figure 10 shows the eddy current loss of coils in transducers with several structural rods. The coil in the transducer with an untreated rod (Figure 10a) exhibited the highest eddy current loss; in comparison to the transducer with the untreated rod, the eddy current loss of the coil in the transducer of the sliced rods (Figure 10d–f) and the transducer of the slit rods (Figure 10b,c) was reduced; and the eddy current loss of the coil in the transducer with the slice treatment rod (Figure 10e) was the smallest.

Taking the transducer of the untreated rod as an example, Table 2 shows the magnetic energy loss of each part of the transducer. The magnetic energy loss of the head mass and tail mass was relatively small, and their losses were ignored. From the table, it can be seen that the Terfenol-D rod has the highest loss, followed by the magnetic conductor and permanent magnet, while the coil has the lowest loss. According to Figure 3, the structure of the Terfenol-D rod has the greatest influence on the vibration of the transducer; the permanent magnet also has a greater impact on the vibration of the transducer, because the permanent magnet adds a bias magnetic field to the Terfenol-D rod to eliminate the frequency doubling phenomenon; and, in addition, the material of the magnetic conductor, the winding type and number of turns of the coil, and the material and shape of the head mass and tail mass have a certain impact on the vibration of the transducer.

Hysteresis loss and eddy current loss are related to frequency and magnetic flux density. The eddy current loss of the rod was reduced after the cutting treatment, the overall magnetic energy loss of the rod was reduced, the magnetic field distribution inside the rod was more uniform, and the output amplitude of the transducer was increased, but the hysteresis loss of the rod was increased. According to the working frequency, the structures of the rod, permanent magnet, and magnetic conductor should be reasonably designed to improve the vibration performance of the transducer.

The inductance of the coil within the transducer can be obtained as follows [23]:

(8) $$L=\frac{\mu_{0}N^{2}S}{l_{\delta }+\frac{l}{\mu_{r}}}$$

where *u*_0_ denotes the vacuum permeability, *N* represents the number of coil turns, *S* stands for the rod’s cross-sectional area, *l* is the rod’s length, and *l_δ_* refers to the air gap length between the rod and the coil, with *u_r_* is the relative permeability.

From Equation (8), it can be seen that the inductance of the transducer is proportional to the permeability, and the permeability indicates the magnetism, magnetic permeability, and magnetization difficulty of the material—the higher the magnetic permeability, the superior the material’s magnetic conductivity.

**Figure 11:**
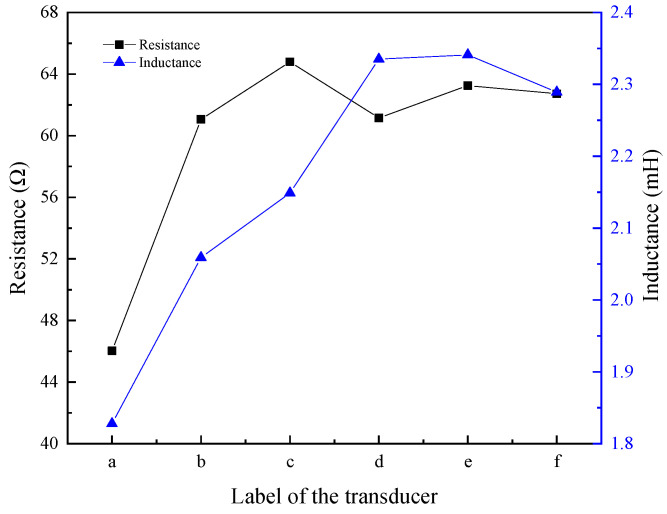
Resistance and inductance of transducers with different structural rods. ((a) Untreated (b) Radial slit (c) Radial cut and bonded (d) Sliced and grooved (e) Slice treatment (f) Sliced at both ends).

Figure 11 shows the inductance and resistance of transducers with different structural rods. For the transducer with an untreated rod, its coil resistance and inductance were the lowest; in comparison with the transducer featuring an untreated rod, both the resistance and inductance of the coil in transducers with sliced rods (Figure 11d–f) and those with slit rods (Figure 11b,c) were increased. The inductance of transducers with sliced rods (Figure 11d–f) was higher than that of those with slit rods (Figure 11b,c). Resistance and inductance indicate the transducer’s ability to dissipate and store magnetic energy. The higher the resistance, the greater the magnetic energy of the transducer; a larger inductance corresponds to a greater amount of magnetic energy stored in the transducer.

## 4. Experimental Test

**Figure 12:**
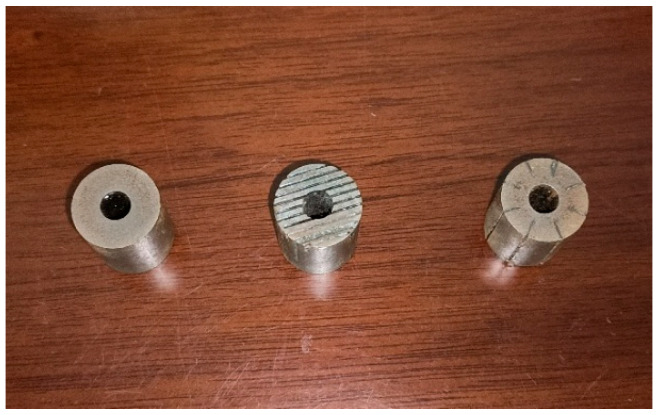
GMM rods with three structures.

**Figure 13:**
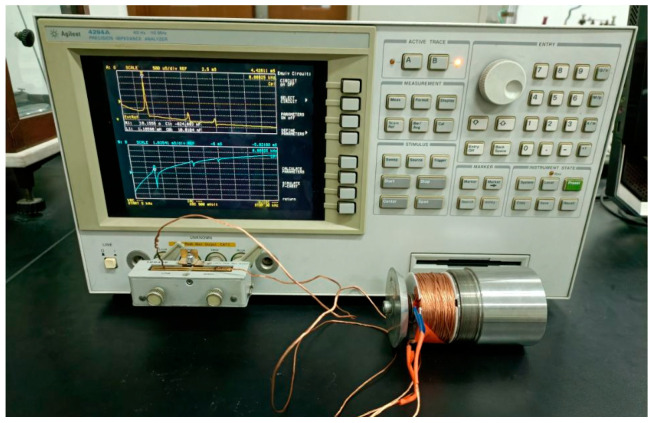
Impedance test of the transducer.

In order to validate the accuracy of the simulation results of giant magnetostrictive transducers with different structural rods, transducers with three structural rods were developed in this works. Figure 12 shows the untreated rod, radial slit rod, and sliced rod at both ends, respectively. Figure 13 shows the impedance test of the transducer. The impedance of the transducers was tested using the impedance analyzer (4294A, Agilent, Santa Clara, CA, USA).

**Figure 14:**
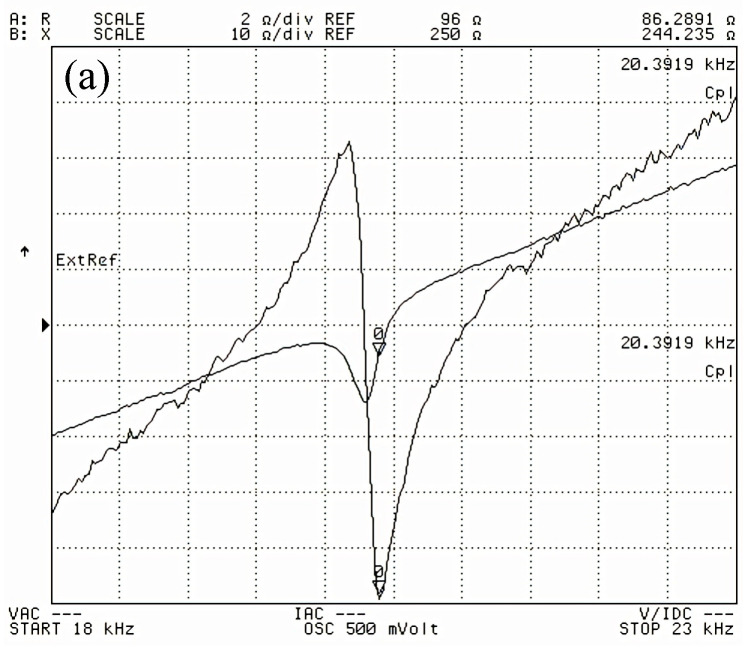

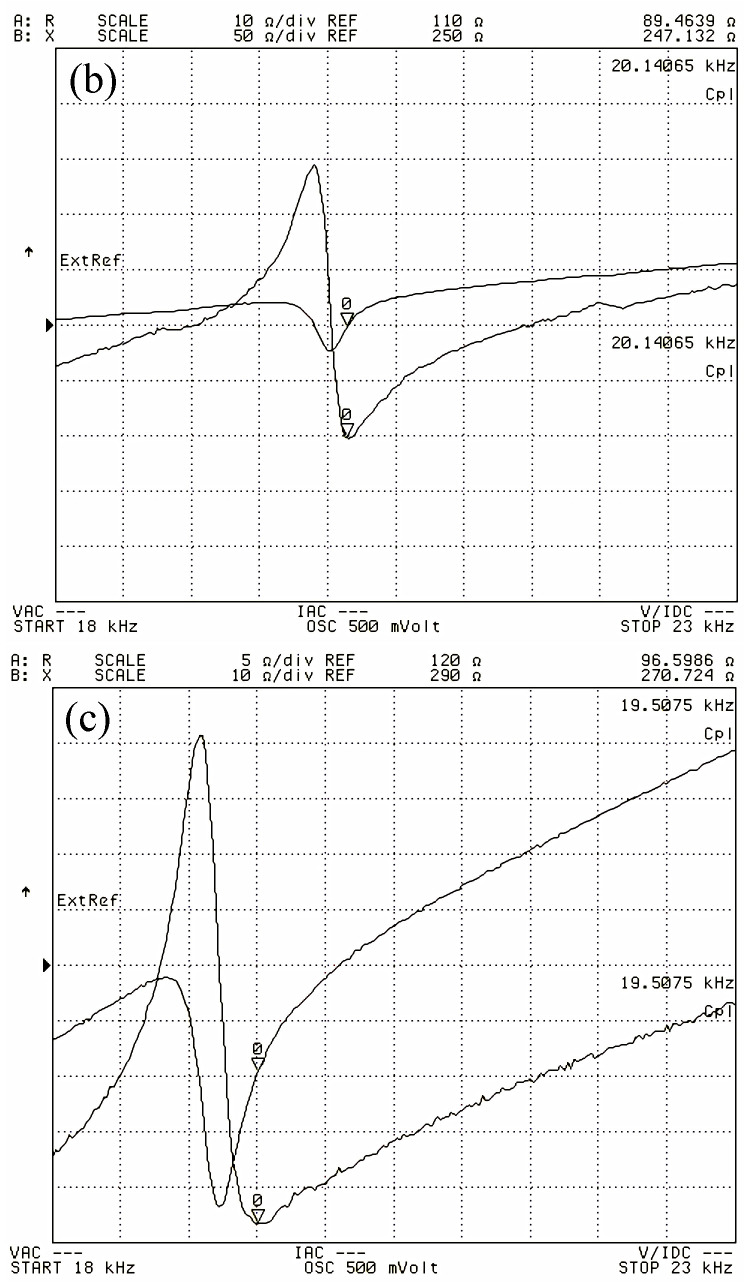
Impedance curve of transducer: (**a**) untreated, (**b**) radial slit, and (**c**) sliced rod at both ends.

Figure 14 shows the tested impedance curves of the three transducers. The resonant frequencies of the three transducers were 20.392 kHz, 20.141 kHz, and 19.508 kHz, respectively, and the relative errors in resonant frequencies of the transducers with the untreated rod, radial slit rod, and sliced rod at both ends calculated by the simulation calculation were 1.20%, 0.37%, and 2.23%, respectively; the experimentally measured values of the resonant frequencies of the three transducers basically agreed with the simulated calculation values.

The resistance and inductance of the three transducers at resonant frequencies were tested. Table 3 shows the simulation calculation values and experimental tests of the resistance and inductance of transducers with three rods. *R_n_* and *R_m_* were the simulated and tested values of the resistance of the transducer, respectively, and *L_n_* and *L_m_* were the simulated and tested values of the inductance of the transducer, respectively. There is a certain error between the calculated values of the finite element and the tested values of the experiment, which may be due to the deviation between the material parameters such as the magnetic permeability of each part of the transducer calculated by the finite element and the actual parameters.

**Figure 15:**
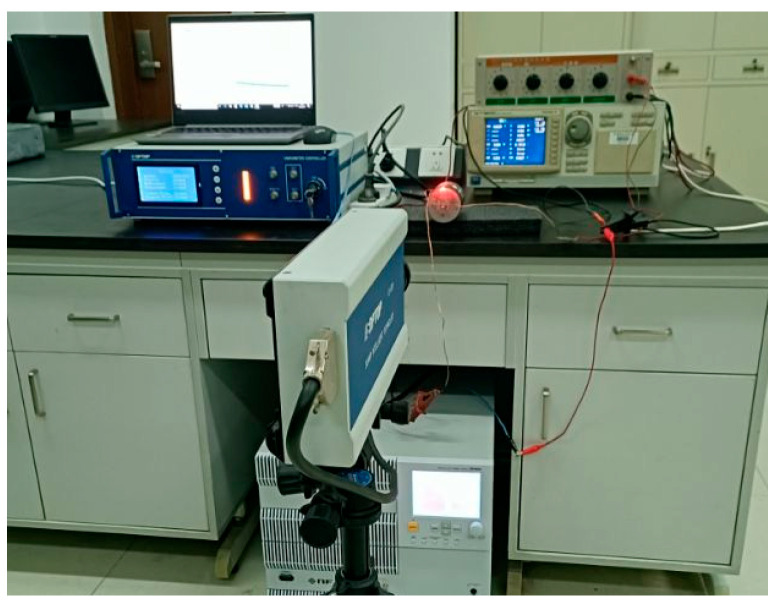
Experimental test.

**Figure 16:**
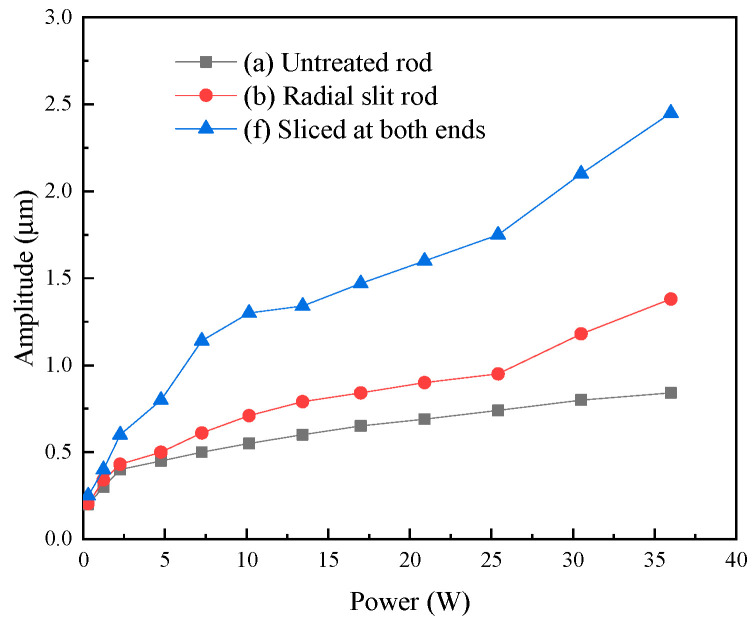
Amplitude of the three transducers.

**Figure 17:**
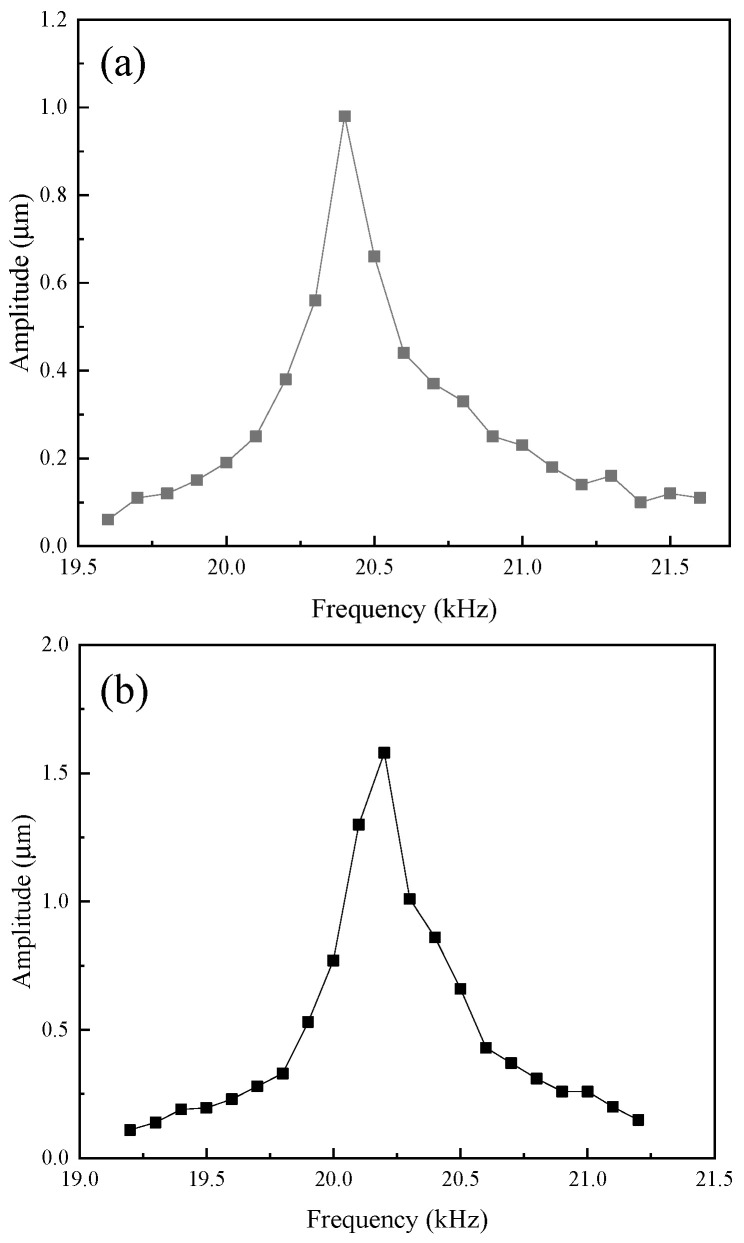

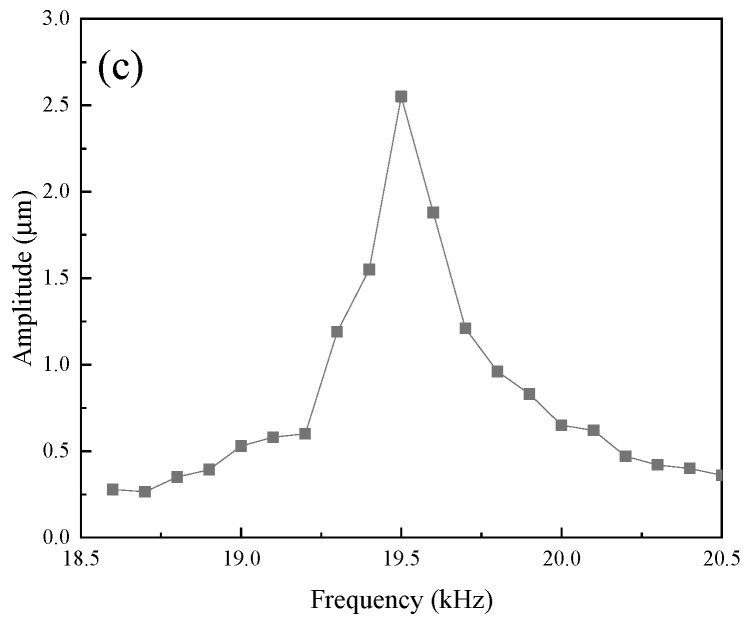
Amplitudes at various frequencies of transducer: (**a**) untreated, (**b**) radial slit, and (**c**) sliced rod at both ends.

A high-speed bipolar power supply (BP4620, NF, Yokohama, Japan) was employed to generate the ultrasonic frequency electrical signal required by the transducer, and a laser vibrometer (LV-S01, Sunny Optical, Ningbo, China) was utilized to measure the transducer’ amplitude. Figure 15 shows the experimental setup for testing the transducer. Figure 16 shows the output amplitudes of three transducers at varying powers. Figure 17 shows the amplitudes at various frequencies of the transducer. The results indicate that the output amplitude of the transducer was largest near the resonant frequency; the amplitudes of all three transducers increased with the increase in power; the transducer with an untreated rod exhibited the smallest output amplitude; and the transducer with a sliced rod at both ends had a larger amplitude than that with a radial slit rod.

## 5. Conclusions

In this paper, finite element software was used to simulate and analyze the giant magnetostrictive transducers with different structures of GMM rods; the resonant frequency, output amplitude, stress distribution, loss, inductance, and resistance of transducers with different structural rods were studied. Transducers with three types of structural rods were developed, and the impedance and output amplitude of the transducers were experimentally tested, and the main conclusions can be obtained:

The peak stress and tangential stress of the rod near one end of the tail mass were greater than those near one end of the head mass; the axial peak stress of the radial slit rod was the smallest, while the axial peak stress of the sliced rod at both ends was the greatest; and the tangential stress of the slice treatment rod was the largest, while that of the untreated rod was the smallest.

The hysteresis loss and eddy current loss were mainly concentrated on the outer diameter surface of the untreated rod, near the slit of the slit rods, and near the connecting part between the slices in the sliced rods. The magnetic conductor, permanent magnet, and coil also have a certain eddy current loss. In the transducer with the untreated rod, the resistance and inductance were the smallest, and inductance of the transducer of the sliced rods were larger than that of the slit rods.

The transducer with an untreated rod featured the highest resonant frequency but the smallest amplitude; transducers with sliced rods had a lower resonant frequency than those with slit rods, whereas their amplitude was greater. The simulated values of the resonant frequency, output amplitude, resistance, and inductance of the transducers of the three structural rods were basically consistent with the tested values.

## Figures and Tables

**Table 1:** Material parameters.

Material	Density (kg/m^3^)	Poisson’s Ratio	Young’s Modulus (GPa)	Conductivity (S/m)	Relative Permeability
Pure iron (DT4C)	7860	0.29	195	1 × 10^6^	1000
Aluminum alloy	2790	0.34	71.5	1 × 10^7^	1
NdFeB	7500	0.24	160	6.25 × 10^5^	1.02
Stainless steel	7900	0.25	195	1.3 × 10^6^	1
GMM	9250	0.3	27.5	1.89 × 10^6^	10.075 + 6.7i

**Table 2:** Magnetic energy loss of the transducer of the untreated rod.

Structural Element	Terfenol-D Rod	Magnetic Conductor	Permanent Magnet	Coil
Loss (W)	6.26	1.59	0.11	0.21

**Table 3:** Resistance and inductance of transducers with different structural rods.

Structure of GMM Rod	*R_n_* (Ω)	*R_m_* (Ω)	*L_n_*/(mH)	*L_m_*/(mH)
(**a**) Untreated	46.030	86.289	1.828	1.907
(**b**) radial slit	61.060	89.464	2.059	1.954
(**c**) Sliced rod at both ends	62.720	96.599	2.289	2.210

## Data Availability

The data that support the findings of this study are available from the corresponding authors upon reasonable request.
